# New Screening Methods in Melanoma

**DOI:** 10.3390/cancers16244186

**Published:** 2024-12-16

**Authors:** Aleksandra Czerw, Andrzej Deptała, Olga Partyka, Monika Pajewska, Anna Badowska-Kozakiewicz, Michał Budzik, Katarzyna Sygit, Zygmunt Kopczyński, Piotr Czarnywojtek, Elżbieta Cipora, Magdalena Konieczny, Tomasz Banaś, Elżbieta Grochans, Szymon Grochans, Anna Maria Cybulska, Daria Schneider-Matyka, Ewa Bandurska, Weronika Ciećko, Jarosław Drobnik, Piotr Pobrotyn, Urszula Grata-Borkowska, Joanna Furtak-Pobrotyn, Aleksandra Sierocka, Michał Marczak, Remigiusz Kozlowski

**Affiliations:** 1 Department of Health Economics and Medical Law, Medical University of Warsaw, 01-445 Warsaw, Poland; 2 Department of Economic and System Analyses, National Institute of Public Health NIH-National Research Institute, 00-791 Warsaw, Poland; 3 Department of Oncology Propaedeutics, Medical University of Warsaw, 01-445 Warsaw, Polandmichal.budzik@wum.edu.pl (M.B.); 4 Faculty of Medicine and Health Sciences, Calisia University, 62-800 Kalisz, Poland; 5 Polytechnic Faculty, Calisia University, 62-800 Kalisz, Poland; 6 Medical Institute, Jan Grodek State University in Sanok, 38-500 Sanok, Poland; 7 Department of Radiotherapy, Maria Sklodowska-Curie Institute-Oncology Center, 31-115 Cracow, Poland; 8 Department of Nursing, Faculty of Health Sciences, Pomeranian Medical University in Szczecin, 71-210 Szczecin, Poland; 9 Department of Pediatric and Oncological Surgery, Urology and Hand Surgery, Faculty of Medicine and Dentistry, Pomeranian Medical University in Szczecin, 71-252 Szczecin, Poland; 10 Center for Competence Development, Integrated Care and e-Health, Medical University of Gdansk, 80-204 Gdansk, Poland; 11 Department of Family Medicine, Faculty of Medicine, Wroclaw Medical University, 51-141 Wroclaw, Poland; 12 Pulsantis Specialist and Rehabilitation Clinic Ltd., 53-238 Wroclaw, Poland; 13 Citodent Dental Center Furtak-Pobrotyn & Company Limited Partnership, 05-220 Olawa, Poland; 14 Department of Management and Logistics in Healthcare, Medical University of Lodz, 90-131 Lodz, Poland; 15 Department of Innovation, Merito University in Poznan, 61-895 Poznan, Poland

**Keywords:** melanoma, MM, screening, clinical trials

## Abstract

Background: The World Health Organisation reports that melanoma had an incidence of 331,722 cases worldwide in 2022, ranking it 17th on the list of the most prevalent malignancies. This disease is a threat to public health as years of potential life lost from melanoma deaths constitute an economic and social burden; it is, however, curable if detected early. This study aims to show current trends in clinical trials for melanoma screening. Materials and Methods: The analysis was conducted using data from clinicaltrials.gov. The analysis considered both interventional and observational studies on melanoma screening. Only the studies with complete and active statutes by 4 September 2024 were included in the analysis. Results: Out of 25 studies registered in clinical trial databases regarding melanoma screening, 20% research advanced imagining techniques employing innovative machine learning algorithms, while 16% explore behavioural interventions. Conclusions: Intensification of behavioural interventions and health promotion activities is recommended.

## Simple Summary

Melanoma is an aggressive type of skin cancer. This type of cancer is invasive but preventable and treatable if detected early. This article explores currently ongoing new trials in melanoma screening. Activities aimed at behavioural changes and health promotion are valuable tools to further reduce the burden of this disease.

## 1. Introduction

The World Health Organisation reports that melanoma had an incidence of 331,722 cases worldwide in 2022, ranking it 17th on the list of the most prevalent malignancies [1]. The age-standardised incidence rate was 3.2/100,000. However, the incidence rate values for Europe and North America are comparatively higher, standing at 10.4/100,000 and 16.3/100,000, respectively, with Oceania reaching an alarming 29.8/100,000 [1,2]. Australia had the highest recorded incidence rate value of 37.0/100,000. In North America, the incidence rate is higher in males (19.0/100,000) compared to females (14.3/100,000) [1,2]. The same pattern of sex differences can be observed in Oceania, where the incidence rate in males is equal to 37.4/100,000, and that in females is equal to 23.1/100,000 [1]. In Europe, the incidence rates for males and females are close to each other, i.e., 11.0/100,000 for males and 10.1/100,000 for females, and in Asia, the incidence rates for both sexes are low, i.e., 0.44/100,000 for males and 0.40/100,000 for females [1]. The difference between females and males that can be observed in the total world population, i.e., 3.7/100,000 for males and 2.9/100,000 for females, is a consequence of the differences observed in North America and Oceania [1].

The global age-standardised mortality rate stood at 0.53/100,000, ranking it 22nd on the list of malignancy-related deaths. Similarly, as with incidence rates, mortality rates are higher for North America, Europe, and Oceania at 0.74/100,000, 1.2/100,000, and 1.7/100,000, respectively, with New Zealand having the highest mortality rate at 3.9/100,000 [1]. In North America, the mortality rate is higher in males (1.5/100,000) compared to females (0.74/100,000). Similarly to incidence rates, the same pattern of sex differences can be observed in Oceania, where the mortality rate in males is equal to 3.0/100,000 and that in females is equal to 1.7/100,000 [1]. In Europe, the mortality rates for females and females are close to each other, i.e., 1.9/100,000 for males and 1.2/100,000 for females, and in Asia, the mortality rates for both sexes are low, i.e., 0.24/100,000 for males and 0.19/100,000 for females [1]. The minor difference between females and males that can be observed in the total world population, i.e., 0.65/100,000 for males and 0.43/100,000 for females, can be mainly attributed to the differences observed in North America and Oceania [1].

Despite decreasing mortality rates overall, incidence has increased over the last few years. This could be attributed to improved detection methods, but it is also crucial to take into account risk factors related to lifestyle [3]. Even though alcohol consumption and smoking have been strongly correlated with an increased risk of developing other cancers, ambivalent results have shown that these factors may be indirectly related to the damage caused by exposure to UV radiation [4]. Review studies also suggest that diet might have an impact on melanoma formation due to interactions between UV exposure and certain foods, such as furocoumarins in citrus fruits [5]. 

Based on data from the European Commission, the reported risk of developing melanoma by age 74 is 1:74 for women and 1:66 for men [2]. Most European countries have a high likelihood of five-year survival, typically ranging from 80% to 90%. However, in some countries like Slovakia, Croatia, Estonia, and Lithuania, the percentage is lower, ranging from 70% to 80%, while in Latvia and Poland, it falls between 60% and 70%. In Bulgaria, it stands at a mere 50% [2].

Risk factors for melanoma include exposure to ultraviolet radiation from sunlight (both intermittent sun exposure and sunbathing), tanning bed lamps, childhood sunburn, having multiple pigmented nevi on the skin (>100), the presence of atypical nevi, a family history of melanoma or skin cancer, a previous diagnosis of melanoma, a previous malignancy (especially thyroid cancer, soft tissue cancer, cervical cancer, and acute myeloid leukemia), a high lifetime intake of spirits, a weakened immune system (e.g., as a result of HIV infection, transplant, Non-Hodgkin’s lymphoma or chronic lymphocytic leukemia), age, fair complexion [6], light eye colour, freckles, and skin phototype II according to the Fitzpatrick scale (i.e., skin that burns easily and is minimally susceptible to tanning), but also skin type I and III when compared to IV. Also included are, CDKN2A, CDK4, BAP1, MITF, TERT, ACD, TERF2IP or POT1 mutation, one or more variants of MC1R, and having blond or red hair [7]. While UV exposure is the main risk factor for melanoma, some studies have provided inconsistent results regarding lifestyle-related risk factors. The risk of melanoma is also higher among those with a genetic condition known as Xeroderma pigmentosum (XP) [8].

Diagnostic and screening methods include (video) dermoscopy, reflectance confocal microscopy (RCM), and skin biopsy.

Preventing melanoma involves limiting exposure to ultraviolet radiation and being mindful of changes in pigmented nevi. A diet rich in fruits, vegetables, nuts, herbs, coffee, and fish also protects against melanoma [9]. Antioxidants, such as vitamins A, D, and E and zinc, might play an important role in reducing the risk of melanoma due to their effects on underlying mechanisms of the disease, including immunomodulatory attributes and the cell growth-inhibitory effects, although the research is not conclusive, because some is based on animal studies and some is epidemiological [10]. Caffeine also shows promising potential for its protective effect against melanoma; its effectiveness was verified in many cohort studies.

The U.S. Preventive Services Task Force (USPSTF) recommends limiting UV exposure for fair-skinned individuals aged 6 months to 24 years and older [11]. Also, older adults with fair complexion should be counselled similarly if additional risk factors besides fair complexion are present. However, the USPSTF concludes that existing evidence suggests that the net benefit of counselling all adults older than 24 years is not sufficient. Therefore, in determining whether counselling is appropriate in individual cases, patients and clinicians should take the presence of risk factors for skin cancer into consideration. Also, according to the USPSTF, on the basis of the research completed so far, it is not possible to adequately verify the balance of benefits and harms of counselling adults about skin self-examination as an effective way to prevent skin cancer.

## 2. Materials and Methods

This study aims to present current trends in clinical trials for melanoma screening.

The analysis was conducted using data from ClinicalTrials.Gov, the world’s largest registry of clinical trials. This registry is maintained by the US National Institute of Health and the National Library of Medicine. ClinicalTrials.Gov is an extensive database that contains information on more than 507,000 clinical trials conducted in over 220 countries worldwide [12]. Given the subject matter covered, the analysis exclusively incorporated studies relevant to melanoma.

The analysis considered both interventional and observational studies on melanoma screening. To present the current research trends, the search period was narrowed to 2019–2024. Only the studies with complete and active statutes by 4 September 2024 were included in the analysis. Also, the studies regarding treatment appeared in the response of out query, because the evaluation of treatment methods’ efficacy needs continuous screening. These studies focused on the effectiveness of modifying the gut microbiome to increase the number of patients responding positively to treatment, the effects of switching from targeted therapy to immunotherapy, the efficacy of neoadjuvant and adjuvant therapy in conjunction with targeted therapy and immunotherapy in reducing the risk of recurrence in patients with advanced melanoma, potentially effective drugs for treating melanoma with lymph node involvement and in-transit metastasis or inoperable and metastatic melanoma, co-administration of drugs, the usefulness of pharmacogenomic tests in developing supportive care, or the efficacy of personalised vaccines in treating patients with melanoma stage III or IV. These studies were also excluded from further analysis.

**Figure 1:**
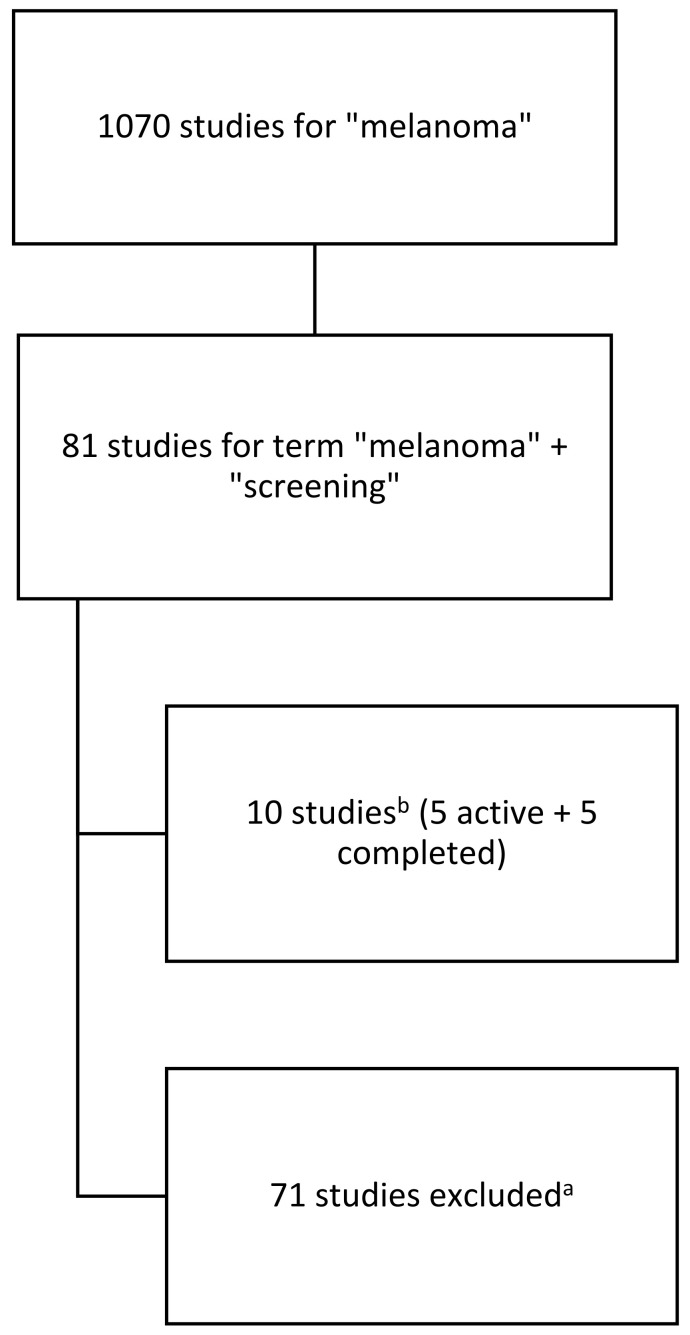
Diagram outlining the search and inclusion of studies in the analysis. a—studies with status: terminated, not yet recruiting, recruiting or other, and studies regarding treatment; b—in the period from 1 September 2019–14 September 2024.

Figure 1 below outlines the process of integrating studies into the analysis.

## 3. Results

Ten studies were analysed further, with five currently active and five terminated.

Table 1 presents the analysis of the melanoma screening studies collected during the study period.

**Figure 2:**
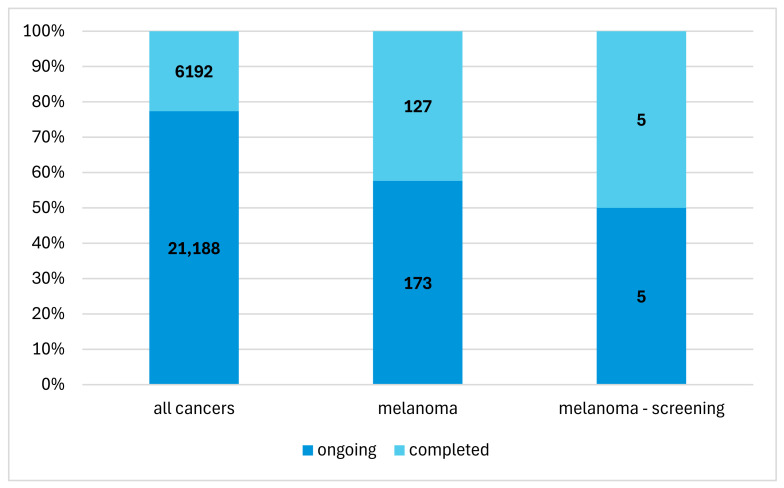
The percentage of melanoma screening studies in the context of overall cancer research.

From 2019 to 2024, the database recorded 27,380 cancer studies. Of this number, melanoma studies collectively accounted for 1.1% (*n* = 300) (Figure 2). Melanoma screening studies constituted 7.6% (*n* = 81) of all melanoma studies.

Out of the ten studies, two were initiated in 2020, six in 2021, and two in 2022. Out of five completed studies, two were completed in 2021, with the other three in 2021, 2022, and in 2023, respectively. Out of five ongoing studies, two were planned to finish in 2025, two were planned to finish in 2026, and one was planned to finish in 2024.

Five studies were intervention studies, and the remaining five were observational. Nine studies exclusively consisted of adult participants. Study sample sizes ranged from 10 to 1000 participants. However, large studies with sample sizes larger than 300 were in the majority. Two studies focused on behavioural interventions; eight examined diagnostic procedures.

The study conducted between 2020 and 2021 investigated the effectiveness of skin self-examination in a cohort of 1000 female patients aged 18–65 (1.). The study participants were randomly split into two groups, i.e., a study group that received educational intervention on skin self-examination and a control group [13]. Women in the study group received an educational booklet with instructions for skin self-examination, including regular assessment of the perimeter around pigmented moles, their colour and diameter, and an observation scoring system. They were also instructed to monitor any changes over time [13]. Women in the control group received an educational booklet on healthy lifestyles, including sleep quality, physical activity, and healthy eating. Both groups received monthly reminders to adhere to the recommendations in the educational booklets. The study lasted 3 months and found that the educational intervention for skin self-examination was effective. The study group had a higher percentage of women who adhered to the recommendations (70.4% in the study group vs. 42.6% in the control group), a higher percentage of women who identified moles of concern (86.0% in the study group vs. 4.9% in the control group), and a slightly higher percentage of women diagnosed with melanoma (2.6% in the study group vs. 1.2% in the control group). The large sample sizes reinforce the conclusions of this study.

In the evaluation conducted in study 2, a targeted educational intervention aimed at parents with a personal history of melanoma and whose children have experienced sunburn in the past 12 months is included [14]. The intervention consists of three educational meetings followed by text messages received quarterly summarising the educational content. Evaluations encompass various factors, including the incidence of sunburn in children, the degree of skin pigmentation resulting from UV exposure, sunscreen usage, the practice of covering the skin with clothing, and intentional sunbathing.

In the initial study on screening diagnosis (3.), the researchers assessed the use of artificial intelligence-based software in processing dermoscopic images [15]. Processing results are intended to assist in biopsy decision-making. The primary focus of the verification pertained to the sensitivity and specificity of diagnoses derived from the software’s recommendations and the correlation with diagnoses obtained from biopsy results. In the subsequent study (4.), clinical total body photography (TBP) of the skin surface of patients at increased risk of melanoma was collected to train artificial intelligence algorithms such as neural networks and random forests in detecting worrying changes in pigmented nevi [16]. By employing artificial intelligence algorithms, the processing of TBP images will be dramatically enhanced, saving considerable time compared to human analysis. The applicability of electrical impedance spectroscopy is also being evaluated (5.) [17]. Currently, data on electrical impedance are being gathered from patients with multiple pigmented nevi (at least 100, with a minimum of 3 having a diameter exceeding 5 mm).

A subsequent study (6.) involved the safety of using and determining appropriate doses of the radiopharmaceuticals Ga-68-PNT6555 and Lu-177-PNT6555 [18].

In a further study (7.), researchers analysed changes in pigmented nevi related to pregnancy. To accomplish this goal, two groups of women were subjected to body scans: one group consisted of pregnant women at least 12 weeks along, while the other group served as the control and consisted of non-pregnant women [19]. Three body scans were performed in the group of pregnant women: one during the 12–16-week period of pregnancy, another during the 29–33-week period, and a third between 8 and 12 weeks after delivery. The control group underwent two body scanning sessions. The scan interval in the control group ranged from 17 to 21 weeks. Pigmented lesions identified in the scan were examined using a machine learning algorithm to determine the likelihood of melanoma development. Additionally, the effectiveness of melanoma detection was assessed by comparing two-dimensional and three-dimensional body imaging (8.). The study was conducted twice, with a 24-month gap between each [20], and it is based on the largest sample if compared to other observational studies included in the current analysis. The results of the biopsies accurately determined the indication of suspicious pigmented nevi. Furthermore, samples were taken from the microbiota of patients diagnosed with melanoma and non-small-cell lung cancer (9.) to determine the characteristics of the microorganisms comprising the microbiota of patients from both groups [21], which can then be used to assess the risk of each of the two types of cancer based on microbiota analyses [21]. The use of an app to collect the medical histories of patients’ families is also under evaluation (10.) [22]. Collected data are then combined with the patient’s existing medical records. Thus, the integration of test results and family history data enables informed decisions on diagnostic and preventive management, resulting in a decline in cancer mortality, particularly melanoma, and mitigation of the burden associated with chronic disease.

## 4. Discussion

Melanoma screening tests primarily involve dermoscopy and skin biopsies. Prophylaxis, on the other hand, mainly comprises limiting exposure to ultraviolet radiation. Nevertheless, the U.S. Preventive Services Task Force (USPSTF) indicates the need for further research in both areas, recognising that the empirical evidence collected to date is insufficient [11]. Hence, additional research is required on the correlation between ultraviolet exposure and melanoma risk and the effectiveness of behavioural interventions to encourage a reduction in ultraviolet exposure. It is crucial to note the scarcity of studies on behavioural interventions, which underscores the urgent need for more research. Specifically, it would be valuable to conduct large-sample studies on controlling sun exposure, sunburn incidence, the occurrence of skin lesions, skin type and age, and adherence to recommendations on protecting oneself from overexposure to the sun. Among the studies registered in ClinicalTrials.Gov, the world’s largest clinical trial repository, only two studies explore behavioural interventions—one focusing on children at risk resulting from two risk factors, a medical history of diagnosed melanoma, and at least one sunburn in the last 12 months, and another study focusing on adult women—while, as explained in the introduction section, according to epidemiological data, if there are differences between males and females, males are more at risk, both in terms of incidence and in terms of mortality.

Also, it seems that studies focused on behavioural interventions could benefit further from theoretical approaches to changes in health behaviours. The transtheoretical model [23] views changes in health behaviours as a series of six stages: the precontemplation stage, the contemplation stage, preparation, the action stage, maintenance, and the termination stage. According to this model, behavioural interventions need to be tailored according to the stage of change that a person is in. The Health Belief Model [24] suggests that patients’ beliefs relating to the effectiveness, ease, and consequences of engaging in or not engaging in a certain health behaviour will determine whether one engages in the behaviour or not. The theory of planned behaviour [25] describes how people decide to behave in a certain way on the basis of their intentions, which are dependent on their attitudes and perceptions of the social norms regarding the behaviour. The studies based on classical theoretical approaches to changes in the health behaviours would definitely be more complex and need repeated measurements, but at the same time, they would take important factors into consideration.

Five studies included in the current review deal with advanced body imaging methods employing machine learning algorithms, like artificial intelligence-based software in processing dermoscopic images, total body photography, and electrical impedance spectroscopy. Two studies look at the feasibility of using additional information sources such as microbiota composition and data from the medical history of patients’ families in conjunction with their current diagnostic test results.

The review presented in our paper has certain limitations. Firstly, most studies presented take into consideration adult patients at risk. Only one study is focused on younger individuals specifically. Secondly, promising results of applying new technologies like imaging and AI-related methods will need to be evaluated in terms of their cost-effectiveness as well. It is possible that their application will lead to quicker patient diagnosis, reduced healthcare costs, and a shift towards more tailored healthcare approaches [26]. However, their development and the organisation of widespread access to patients will definitely need investment first.

Also, it is worth noting that only three studies presented are longitudinal studies, and in general, longitudinal studies lead to stronger conclusions in comparison to cross-sectional studies.

Molecular diagnostics is another approach that was not present in the studies acquired in the response to our query. The tests are in the early stages of research, but seem to be promising [27,28,29].

Awareness of melanoma in Poland is disseminated through the melanoma prevention programme “Znamię! Znam je?” (“Nevi! Do I know them?”) [30] implemented by units of the State Sanitary Inspectorate. This programme is addressed to secondary school students, their parents, and school teaching staff. It covers topics like skin self-examination, protective factors, and groups at risk of melanoma and it is under constant evaluation within the national health programme. Educational campaigns are also carried out during the annual Melanoma Awareness Week. In addition, patients are urged to examine pigmented nevi as part of the ‘Planuję długie życie’ (I plan to live a long life) campaign conducted by the Ministry of Health within the framework of the National Oncology Strategy for 2020–2030, comprising health education, health promotion, and prevention [31]. The education campaign regarding melanoma provides interactive video materials aimed at young people.

## 5. Conclusions

Melanoma is an aggressive type of skin cancer; however, it is curable if detected early. Early detection and prevention are crucial; therefore, studies involving new diagnostic and detection tools are necessary. Out of 10 studies registered in the ClinicalTrials.Gov database regarding melanoma screening, 60% research advanced imagining techniques employing innovative machine learning algorithms, while 16% explore behavioural interventions. The intensification of behavioural interventions and health promotion activities aimed at melanoma prevention and lifestyle changes is recommended, especially if connected to more complex theoretical approaches explaining how such a change in expected health behaviours may be achieved.

## Figures and Tables

**Table 1:** Distribution of clinical trial analysis results in the ClinicalTrials.Gov repository according to adopted categories.

No.	Group	Title	Years	*n*	Status	Study	Population
1.	Interventions	Targeted Melanoma Detection With Skin Self-Examination During COVID-19 Restricted Physician Access	2020–2021	1000	completed	interventional	adult women
2.	behavioural	Family Lifestyles, Actions, and Risk Education Intervention: Version 2	2020–2025	762	ongoing	interventional	diad of a parent with a medical history of diagnosed melanoma and a child with at least one sunburn in the last 12 months
3.	Diagnosing	Diagnostic Precision of the AI Tool Dermalyzer to Identify Malignant Melanomas in Subjects Seeking Primary Care for Melanoma-suspected Cutaneous Lesions	2022–2023	250	completed	observational	adults with at least one skin lesion requiring a diagnosis
4.		MoleGazer Development Feasibility Study	2021–2026	374	ongoing	interventional	adults aged 18–80 years with an increased risk of melanoma
5.		Study of the Nevisense Device to Assess Atypical Skin Lesions	2021–2025	40	ongoing	observational	adults aged 30 and over with at least 100 pigmented nevi
6.		FAPi Radioligand OpeN-Label, Phase 1 Study to Evaluate Safety, Tolerability, and DosImetry of [Lu-177]-PNT6555; A Dose Escalation Study for Treatment of Patients With Select Solid Tumors (FRONTIER)	2022–2026	10	ongoing	interventional	adult cancer patients with metastatic cancer
7.		Pregnancy-related Changes in Melanocytic Nevi	2021	50	completed	observational	women aged 18–40
8.		Melanoma Detection in Switzerland With VECTRA	2021–2024	455	completed	observational	adults at increased risk of melanoma
9.		Establishment of the Human Intestinal and Salivary Microbiota Biobank-Oncologic Diseases	2021–2022	50	completed	observational	adults diagnosed with melanoma or non-small-cell lung cancer
10.		Family History App in Personalized Medicine	2021–2024	627	ongoing	interventional	adults aged 30–69

## References

[1] Ferlay J., Ervik M., Lam F., Laversanne M., Colombet M., Mery L., Piñeros M., Znaor A., Soerjomataram I., Bray F. (2024). Global Cancer Observatory: Cancer Today. International Agency for Research on Cancer: Lyon, France. https://gco.iarc.who.int/today.

[2] European Commission Skin Melanoma Burden in EU-27. https://publications.jrc.ec.europa.eu/repository/handle/JRC125928.

[3] Wunderlich K., Suppa M., Gandini S., Lipski J., White J.M., Del Marmol V. (2024). Risk Factors and Innovations in Risk Assessment for Melanoma, Basal Cell Carcinoma, and Squamous Cell Carcinoma. Cancers.

[4] Rivera A., Nan H., Li T., Qureshi A., Cho E. (2016). Alcohol Intake and Risk of Incident Melanoma: A Pooled Analysis of Three Prospective Studies in the United States. Cancer Epidemiol. Biomark. Prev..

[5] Marley A.R., Li M., Champion V.L., Song Y., Han J., Li X. (2021). The association between citrus consumption and melanoma risk in the UK Biobank. Br. J. Dermatol..

[6] American Cancer Society Risk Factors for Melanoma Skin Cancer. https://www.cancer.org/cancer/types/melanoma-skin-cancer/causes-risks-prevention/risk-factors.html.

[7] Lock-Andersen J., Drzewiecki K.T., Wulf H.C. (1999). Eye and hair colour, skin type and constitutive skin pigmentation as risk factors for basal cell carcinoma and cutaneous malignant melanoma. A Danish case-control study. Acta Derm. Venereol..

[8] Brambullo T., Colonna M.R., Vindigni V., Piaserico S., Masciopinto G., Galeano M., Costa A.L., Bassetto F. (2022). Xeroderma Pigmentosum: A Genetic Condition Skin Cancer Correlated-A Systematic Review. Biomed Res. Int..

[9] Fortes C., Mastroeni S., Levati L., Alotto M., Ricci F., D’Atri S. (2024). The potential impact of dietary choices on melanoma risk: An anti-inflammatory diet. Genes. Nutr..

[10] Dong Y., Wei J., Yang F., Qu Y., Huang J., Shi D. (2023). Nutrient-Based Approaches for Melanoma: Prevention and Therapeutic Insights. Nutrients.

[11] US Preventive Services Task Force Prostate Cancer: Screening. https://www.uspreventiveservicestaskforce.org/uspstf/recommendation/skin-cancer-counseling.

[12] National Library of Medicine, ClinicalTrails.gov. https://clinicaltrials.gov/.

[13] Robinson J.K., Wahood S., Ly S., Kirk J., Yoon J., Sterritt J., Gray E., Kwasny M. (2021). Melanoma detection by skin self-examination targeting at-risk women: A randomized controlled trial with telemedicine support for concerning moles. Prev. Med. Rep..

[14] Wu Y.P., Stump T.K., Hay J.L., Aspinwall L.G., Boucher K.M., Deboeck P.R., Grossman D., Mooney K., Leachman S.A., Smith K.R. (2023). The Family Lifestyles, Actions and Risk Education (FLARE) study: Protocol for a randomized controlled trial of a sun protection intervention for children of melanoma survivors. Contemp. Clin. Trials.

[15] https://clinicaltrials.gov/study/NCT05172232.

[16] https://clinicaltrials.gov/study/NCT05015816?cond=MoleGazer%20Development%20Feasibility%20Study&rank=1.

[17] https://clinicaltrials.gov/study/NCT04705168?cond=Study%20of%20the%20Nevisense%20Device%20to%20Assess%20Atypical%20Skin%20Lesions&rank=1&tab=table.

[18] https://clinicaltrials.gov/study/NCT05432193?cond=FAPi%20Radioligand%20OpeN-Label,%20Phase%201%20Study%20to%20Evaluate%20Safety,%20Tolerability%20(FRONTIER)&rank=1&tab=table.

[19] https://clinicaltrials.gov/study/NCT05148455?cond=Pregnancy-related%20Changes%20in%20Melanocytic%20Nevi&rank=1.

[20] https://clinicaltrials.gov/study/NCT04605822?cond=Melanoma%20Detection%20in%20Switzerland%20With%20VECTRA&rank=1.

[21] https://clinicaltrials.gov/study/NCT04698161?cond=Establishment%20of%20the%20Human%20Intestinal%20and%20Salivary%20Microbiota%20Biobank%20-%20Oncologic%20Diseases&rank=1&tab=table.

[22] https://clinicaltrials.gov/study/NCT04726319?cond=Family%20History%20App%20in%20Personalized%20Medicine&rank=1&tab=table.

[23] Prochaska J.O., Redding C.A., Evers K.E., Glanz K., Rimer B.K., Viswanath K. (2015). The transtheoretical model and stages of change. Health Behavior: Theory, Research, and Practice.

[24] Ajzen I. (1988). Attitudes, Personality, and Behavior.

[25] Skinner C.S., Tiro J., Champion V.L., Glanz K., Rimer B.K., Viswanath K. (2015). The health belief model. Health Behavior: Theory, Research, and Practice.

[26] Gandolfi G., Dallaglio K., Longo C., Moscarella E., Lallas A., Alfano R., Argenziano G., Ciarrocchi A. (2024). AI in diagnostic imaging: Revolutionising accuracy and efficiency. Comput. Methods Programs Biomed. Update.

[27] Gandolfi G., Dallaglio K., Longo C., Moscarella E., Lallas A., Alfano R., Argenziano G., Ciarrocchi A. (2016). Contemporary and potential future molecular diagnosis of melanoma. Expert. Rev. Mol. Diagn..

[28] Knackstedt R.W., Knackstedt T., Gastman B. (2018). Gene Expression Profiling in Melanoma: Past Results and Future Potential. Future Oncol..

[29] Sondak V. (2020). The use of molecular testing at a diagnosis of melanoma. Clin. Adv. Hematol. Oncol. HO.

[30] https://www.gov.pl/web/wsse-warszawa/oksipz-znamie-znam-je.

[31] https://planujedlugiezycie.pl/.

